# Antimicrobial Use and Antimicrobial Resistance: A Population Perspective

**DOI:** 10.3201/eid0804.010312

**Published:** 2002-04

**Authors:** Marc Lipsitch, Matthew H. Samore

**Affiliations:** * Harvard School of Public Health, Boston, Massachusetts, USA; † University of Utah School of Medicine, Salt Lake City, Utah, USA

**Keywords:** Antimicrobial resistance, population dynamics, mathematical model, infectious disease transmission, ecological study, epidemiologic methods

## Abstract

The need to stem the growing problem of antimicrobial resistance has prompted multiple, sometimes conflicting, calls for changes in the use of antimicrobial agents. One source of disagreement concerns the major mechanisms by which antibiotics select resistant strains. For infections like tuberculosis, in which resistance can emerge in treated hosts through mutation, prevention of antimicrobial resistance in individual hosts is a primary method of preventing the spread of resistant organisms in the community. By contrast, for many other important resistant pathogens, such as penicillin-resistant *Streptococcus* *pneumoniae*, methicillin-resistant *Staphylococcus aureus*, and vancomycin-resistant *Enterococcus faecium* resistance is mediated by the acquisition of genes or gene fragments by horizontal transfer; resistance in the treated host is a relatively rare event. For these organisms, indirect, population-level mechanisms of selection account for the increase in the prevalence of resistance. These mechanisms can operate even when treatment has a modest, or even negative, effect on an individual host’s colonization with resistant organisms.

The growth of antimicrobial resistance has prompted calls to reduce unnecessary antibiotic use and to improve treatment protocols to maximize the lifespan of these drugs. These calls rest on the well-supported idea that the use of antimicrobial agents is a powerful selective force that promotes the emergence of resistant strains.

To reduce antimicrobial resistance, multiple, and often conflicting recommendations, have been made. For example, strategies to minimize the burden of resistance in hospitals have included reduction of all antimicrobial classes, increased use of prophylactic antimicrobials to reduce colonization, rotation of different antibiotic classes in a temporal sequence, and simultaneous use of different antimicrobials for different patients (*1*–*6*).

Underlying these often varying recommendations for improving antimicrobial use is frequently conflicting evidence about the relationship between antibiotic treatment and antibiotic resistance. In some pathogens, showing that antibiotic treatment puts treated persons at a greater risk for acquiring resistant organisms has been difficult (*7*,*8*); nonetheless, the cumulative effect of using these antibiotics has clearly been to increase the prevalence of resistance in the population as a whole.

For many pathogens of current concern, especially organisms for which asymptomatic colonization typically precedes infection (e.g., Streptococcus pneumoniae, Staphylococcus aureus, Enterococcus spp., and the gram-negative enteric bacteria), the relationship between antimicrobial use and resistance differs in fundamental ways from the relationship found in Mycobacterium tuberculosis, for which many modern principles of chemotherapy were developed. Furthermore, we argue that the selective effects of antibiotic use on these organisms are poorly understood, and we make specific suggestions for studies that could improve understanding of the mechanisms by which antibiotics exert natural selection on these organisms. Such an understanding will be crucial for the design of rational policies of antibiotic use to maximize the lifespan of existing drugs and to minimize the impact of resistant infections.

## Resistance in People and Populations

Ehrlich's advice that treatment of infections should "hit hard and hit early," formulated in the earliest days of antimicrobial chemotherapy, presciently summarized the principles of treatment for infections such as tuberculosis (TB) (*9*). These principles are embodied in modern protocols of directly observed, short-course chemotherapy, where the goal is to treat with adequate concentrations of multiple drugs and maintain treatment until the bacterial population is extinct.Resistance to each of the major antituberculosis drugs is mediated by single point mutation; therefore tuberculosis treatment is designed to prevent the ascent of subpopulations of mutant bacilli that are resistant to any one of the drugs. Similar principles have been suggested for other infections in which resistance can arise by simple mutation, most notably HIV (*10*), although there has been some controversy on this topic (*11*). In these infections, the relationship between treatment, resistance in the treated person, and resistance in the community at large is relatively clear. Inadequate therapy (owing to subtherapeutic drug concentrations, too few drugs, or poor adherence to therapy) results in the emergence of resistance, and possibly treatment failure, in the treated host. Following the emergence of resistance in the treated host, resistant infections may be transmitted to others (Figure, A; Table).

**Figure:**
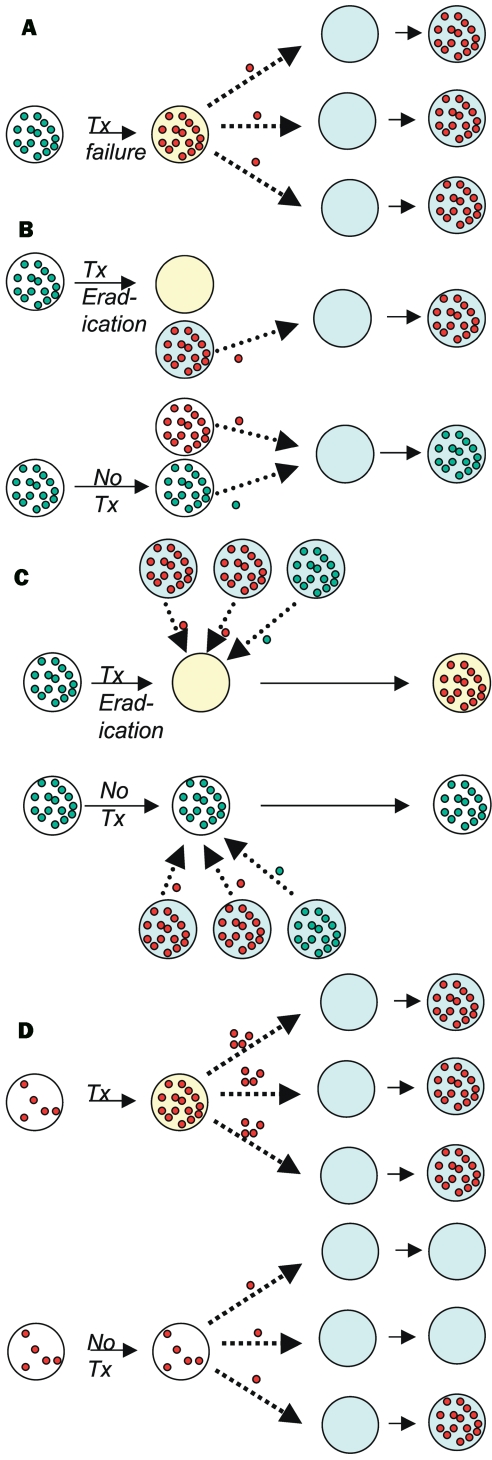
Four mechanisms by which antibiotic treatment can create selection for resistance in the population, showing direct effects—increased resistance in treated (yellow) vs. untreated (white) hosts, and indirect effects—increased resistance in others (turquoise) due to treatment of specific hosts. **(A)** Subpopulations (usually mutants) of resistant (red) bacteria are present in a host infected with a predominantly susceptible (green) strain; treatment fails, resulting in outgrowth of the resistant subpopulation, which can then be transmitted to other, susceptible hosts (turquoise). **(B)** Successful treatment of an individual infected with a susceptible strain reduces the ability of that host to transmit the infection to other susceptible hosts, making those hosts more likely to be infected by resistant pathogens than they would otherwise have been, and shifting the competitive balance toward resistant infections. **(C)** Treatment of an infection eradicates a population of susceptible bacteria carried (often commensally) by the host, making that host more susceptible to acquisition of a new strain. If the newly acquired strain has a high probability of being resistant (as in the context of an outbreak of a resistant strain), this can significantly increase the treated individual’s risk of carrying a resistant strain, relative to an untreated one. **(D)** Treatment of an infection in an individual who is already colonized (commensally) with resistant organisms may result in increased load of those organisms if competing flora (perhaps of another species) are inhibited—leading to increased shedding of the resistant organism and possibly to increased individual risk of infection with the resistant organism.

**Table:** Mechanisms by which antimicrobial treatment has direct and indirect effects on resistance

Mechanism (effect of treatment)	Relationship between selection for resistance and treatment success	Relationship between no./dose of antibiotics and selection	Examples	Figure
mergence of resistance during treatment (D^a^, I^b^)	(−)^c^	(−)	TB, HIV, Pseudomonas aeruginosa, Enterobacter spp.	1a
Reduced transmission of susceptible strains (I)	(+)^d^	(+)	May occur for nearly every infection	1b
Increased susceptibility to colonization (D, I,)	?^e^	?	Commensals of skin, intestinal and respiratory tracts	1c
Increased density of colonization in individuals already colonized with resistant organisms, by inhibiting competitors (D,I)	?	(+)−?	VRE^f^ and antianaerobic treatments	1d

^a^D=direct. ^b^I=indirect. ^c^(-)=inverse relationship. ^d^(+)=positive relationship. ^e^?=relationship uncertain. ^f^VRE=vancomycin-resistant *Enterococci*.

For many pathogens, both the genetics and the epidemiology of resistance differ from those of TB in important ways. For example, methicillin resistance in S. aureus and vancomycin resistance in Enterococcus are mediated by the acquisition of one or several new genes, rather than by point mutations in existing genes. In Streptococcus pneumoniae, penicillin resistance occurs when segments of wild-type penicillin-binding protein genes are replaced with alleles whose sequences differ from the wild-type at multiple positions. These new resistance mechanisms arose and spread in large populations under conditions of antibiotic selection pressure, but they are unlikely to occur de novo in any single person because of the multiple changes involved. Organisms (or plasmids) bearing these types of resistance must be acquired, generally as a consequence of cross-transmission. Furthermore, most of these organisms are not obligate pathogens such as HIV or TB; as a result, much of their exposure to antibiotics occurs during treatment directed at infections caused by other, unrelated organisms.

Because of these genetic and epidemiologic differences, the paradigm for tuberculosis treatment, minimizing resistance in the treated host and the community by preventing the emergence of resistant subpopulations during treatment, is often inapplicable to these organisms (*12*). Antibiotic treatment promotes the spread of these organisms, as suggested by the rapid increases in resistance in many of the organisms after the new drug classes are introduced. However, the effects of treatment in promoting resistance occur by less direct mechanisms, which depend on competitive interactions between drug-resistant and drug-susceptible strains.

## Indirect Effects on Resistance

For any infectious disease, the infection or colonization status of any one (index) patient affects the risk of infection or colonization of others. Measures (such as vaccination or antibiotic treatment) that change the incidence or duration of infection in one person will affect that person's contacts (*13**-**14*). Just as vaccination programs benefit those who are not vaccinated because of the phenomenon of herd immunity, antibiotic usage by some persons may increase the risk of colonization or infection with resistant organisms in people who have not received antibiotics. Members of a population experience indirect effects of antimicrobial use, defined as the enhancement of risk for acquiring a resistant organism, because of the use of antimicrobials by other persons in the group or population.

For example, simply by eradicating susceptible organisms, and thereby reducing the opportunities for transmission of susceptible strains, antibiotics received by treated hosts can increase the probability that other hosts will acquire resistant variants (Figure, B; Table). For many pathogens, acquisition of one strain reduces a person's chances of acquiring other strains, either via immune responses, via direct interference (*15*–*17*), or both. These inhibitory interactions create competition between resistant and susceptible strains. As a result, treatment of some patients, by eradicating susceptible strains and thereby reducing their ability to transmit to other hosts, is advantageous to resistant strains in the population. Mathematical models (*18*–*22*) and epidemiologic studies (*23*) suggest that this mechanism of shifting the competitive balance in favor of resistant strains can increase the prevalence of resistant organisms in the community, alone or in combination with other mechanisms. An important feature of this kind of indirect effect is that it need not involve an increase in a patient’s own risk of carrying resistant organisms, only a reduction in the duration or probability of carrying susceptible ones.

In these organisms, the increase in transmission of resistant pathogens is a consequence of successful treatment of the infected host, resulting in the eradication of drug-susceptible pathogens that colonize or infect that host. As a consequence, the more effective a treatment is at eradicating drug-susceptible populations of these organisms, the more it will promote the spread of resistant ones. This spread contrasts with TB, in which treatment failure is often associated with the emergence of resistance in treated hosts, so unsuccessful treatment is seen as a factor promoting the spread of resistance (although, over a time scale of decades, this type of indirect mechanism described here may play a role even in tuberculosis [*21*]).

## Combinations of Direct and Indirect Effects on Resistance

A third mechanism by which antimicrobial use increases the number of patients colonized or infected with resistant organisms is by modifying the treated host’s colonization resistance (Figure, C; Table). Eradication or reduction of drug-susceptible normal flora by antibiotic treatment may increase vulnerability to acquisition of new strains. This effect will increase the patient’s probability of being colonized with a resistant organism if, during or shortly after treatment, he or she is exposed to others with resistant organisms. This mechanism is direct in the sense that it increases the treated patient’s risk of colonization with resistant organisms but is also associated with indirect effects because of the requirement for transmission. An index host given antibiotics is placed at greater risk for colonization with resistant organisms (direct effect), but this risk is amplified by his or her exposure to other patients harboring resistant organisms, which is in turn enhanced by their use of antibiotics (indirect effect).

A fourth mechanism by which antimicrobial use increases antimicrobial resistance is by increasing the density of resistant organisms within a patient who already harbors such organisms at a lower density (Figure, D; Table). Enhanced shedding of these organisms, resulting in an increased risk to other patients (an indirect effect), has been documented (i.e., in the case of anti-anaerobic agents that increase shedding of vancomycin-resistant Enterococci (VRE) (*24*). An increased risk of resistant infection to the treated patient (a direct effect) may occur if a higher density of resistant organisms places the patient at higher risk of infection with his or her own flora. Unlike the other three ways by which antimicrobial use promotes resistance, this mechanism is mediated through antimicrobial treatment of patients already colonized with the resistant organism.

There are a number of other cases in which direct and indirect effects of antibiotic treatment are combined. Due to the diversity of genetic mechanisms of resistance, the risk of emergence of resistance during treatment represents a continuum, with TB at one end and VRE (or MRSA) at the other. Fluoroquinolone resistance in S. pneumoniae mediated by the accumulation of mutations in the DNA gyrase and topoisomerase IV genes (*25*), or resistance to third-generation cephalosporins in Enterobacteriaceae mediated by mutations in TEM and SHV beta-lactamases located on plasmids (*26*), lie between these two extremes. In these cases, multiple mutations are required to turn a fully susceptible strain into a clinically resistant one. For a patient colonized or infected with a fully susceptible strain, emergence of resistance during treatment may be highly unlikely because of the requirement for selecting multiple mutations. However, in such cases, there may be selection in consecutive hosts for small increases in levels of resistance to a particular compound, resulting eventually in the emergence of clinical resistance (*27*). Patients may be colonized with a mixed flora of resistant and susceptible organisms, and eradication of the drug-susceptible flora may permit outgrowth of the resistant subpopulation (*28*). This mechanism has some formal similarity to what occurs in TB, except that for a colonizing bacterium such as the pneumococcus or the enteric colonizers, outgrowth of resistant organisms in the site of colonization need not be associated with treatment failure. In these cases, the treated patient is at increased risk of carrying resistant organisms (direct effect), but an indirect effect on the population occurs because the treated patient no longer carries susceptible organisms and is, therefore, unable to transmit them.

Treatment with one antimicrobial drug can select for resistance to a number of other, unrelated agents, by several means. If individual organisms are resistant to multiple drugs, then use of any one of these may promote resistance to others (*29*). Additionally, by altering the balance of different components of the indigenous microbial flora, treatment with one agent may increase the load of a pathogen resistant to another agent, simply by killing off competing flora of different species; this has been observed, for example, with anti-anaerobic treatments that increase the load of VRE (*24*). These complexities increase the number of relationships that need to be studied in assessing the effects of antimicrobial use on resistance and also the number of potential confounders in any study.

## Implications for Evaluating Treatment Strategies

Variation in mechanisms of resistance has implications for the choice of antimicrobial therapy and the evaluation of strategies to minimize resistance. Adopting the individual and population-level perspective informs therapeutic decision-making, clinical study design, and public policy.

In TB, preventing the emergence of resistance in a treated host is a sound policy for averting the emergence of resistance at the population level as well (although once resistant strains have emerged, special measures are required to contain them [*30*]). With respect to antimicrobial resistance, what is good for the patient is good for the population.

In contrast, for other types of resistance, antimicrobial treatment may exert individual-level effects that are substantially different in magnitude or even opposite in direction to that of population-level effects. Treatment with a beta-lactam may produce only a small, short-lived increase in the treated patient's odds of carrying or being infected by a resistant pneumococcus (*7*). In some cases, treatment may actually eradicate carriage of a resistant organism, thereby reducing the individual’s risk of resistant carriage. Small or unobservable effects on individual risk have been observed in other cases as well, such as vancomycin use for VRE (*8*,*31*) and the use of various antibiotics for infections with resitant gram-negative rods (*32*). In these cases, preventing resistance in the treated patient may not be the central goal of a prudent antibiotic use policy; instead, treatment should seek to minimize the advantage it provides to resistant organisms in the community or the hospital as a whole, subject to the constraint of providing effective treatment for the patient.

The considerations of the distinctive biologic and epidemiologic mechanisms of antibiotic resistance in different pathogens lead to several broad suggestions for future studies. First, the optimal study design to estimate individual-level effects of antibiotics on colonizing organisms such as VRE and beta-lactam resistant S. pneumoniae is to measure acquisition and loss rates in an observational cohort or experimental study where subjects are serially cultured before, during, and after antibiotic therapy (*23*,*33*). Time-to-event statistical models (e.g., Cox proportional hazards regression) are appropriate analytic methods for these kinds of studies (*23*,*31*,*34*). This design allows investigators to distinguish between the effects of antimicrobials on the risk for acquisition (colonization) and their effects on the risk for clinical infection once an patient has been colonized with a resistant organism.

As a consequence of the mechanisms we have described, the magnitude of an antibiotic’s effect on a patient’s risk of resistant colonization or infection may be dependent on his or her exposure to potential transmission of resistant organisms (*14*). Stated differently, the frequency of contact with others carrying the resistant organisms is likely an important effect-modifier of antibiotic effects for pathogens that do not follow the simple model of emergence of resistance exhibited by organisms such as M. tuberculosis. Individual-level antibiotic effects mediated by alterations in colonization resistance or killing of susceptible bacteria may be greater in settings of high exposure to resistant organisms, for example, during outbreaks (*7*). Controlling for transmission risk or measuring effects conditional on a specified level of transmission risk is advised, when possible. Standard analytic approaches make the assumption that outcomes in different subject are independent, but this assumption is violated in the case of infectious diseases. Use of one of these strategies to model exposure to transmission will help to account for this non-independence of outcomes in different persons in the same study (*13*,*35*–*37*).

One practical result of quantifying direct, individual-level antibiotic effects is to provide information on the short-term risk of infection with a resistant organism to a person about to initiate antibiotic treatment. This hazard needs to be taken into account when weighing the risks and benefits of use of antimicrobial agents in individual patients. However, analogous to the evaluation of vaccine programs, combined direct and indirect antibiotic effects carry increased importance from the public health and policy management perspective (*38*,*39*). The measurement of population-level effects of antimicrobials also has educational value in demonstrating to clinicians and patients the extent to which individual antibiotic use choices have negative consequences for the population as a whole. Such a conflict between individual benefit and the population’s harm is an example of what economists term an “externality” or what environmentalists have called the ‘Tragedy of the Commons” (*40*).

To estimate overall antibiotic effects from data requires group-level studies. Observational group-level studies may lack sufficient data to avoid confounding and other causes of ecologic bias (*41*). For this reason, studies that estimate the effects of individual- and group-level antimicrobial use are generally preferable to ones that contain group-level data alone. Depending on the context, the appropriate group(s) may include the family, the community, the hospital, or the hospital unit or department (*42*–*44*). Further research is necessary to evaluate hierarchical regression methods and compare results obtained from different levels of analysis (*44*).

For the most accurate measurement of overall antibiotic effect on resistance in communities, a cluster-randomized intervention trial is appropriate (*45*). In cluster-randomized trials, the unit of randomization is a group such as a community or a hospital, and multiple units (sometimes as few as six, but often more) are assigned to each of two (or more) treatment arms. We are not aware of published studies using this design to evaluate antibiotic resistance, although we know of two in progress (R. Platt, pers. comm.) (*12*). However, this design has been used in other areas of infectious disease epidemiology for which group level effects are important (such as vaccination programs), and it is considered the standard design for investigations of the effects of insecticide-impregnated bednets in preventing malaria (*45*–*47*). In the context of antimicrobial resistance, cluster-randomized trials have two key advantages. First, unlike studies that gather individual-level data alone, they provide the opportunity to observe the indirect effects of treatment on resistance. Second, they provide a clean way to avoid the statistical problems of nonindependence between patients in a study that may reduce the power or increase the false-positive rate of observational studies. In cluster-randomized studies of antimicrobial resistance, both the incidence rate of infection with resistant organisms in the population and the ratio of resistant to susceptible (or proportion of total organisms that are resistant) would be appropriate study endpoints.

## Role of Mathematical Models

Transmission-dynamic modeling can also play an important role in bridging the gap between individual- and group-level effects (*20*,*21*,*48*–*50*). These models take information about individual-level effects as parameters and make predictions about the response of the population to changes in such parameters as transmission risk or antibiotic usage. Although models cannot substitute for empirical intervention studies, they can be particularly valuable in at least four ways: 1) generating hypotheses about the relationship between antibiotic use and resistance that can be used in designing and prioritizing empirical studies; 2) defining the conditions under which a particular intervention is likely to work, thereby suggesting how empirical results can (and cannot) be extrapolated to other settings; 3) providing explanations for phenomena that have been observed but whose causes were uncertain; and 4) identifying biological mechanisms that, while important, remain poorly understood.

An example of models for generating hypotheses comes from the question of antimicrobial rotation or “cycling.” Cycling of antimicrobial classes in hospitals has been suggested and is currently being evaluated for its ability to curtail resistance in major nosocomial pathogens (*5*,*51*–*54*). One mathematical model of this process has suggested that using a mixture of different drug classes simultaneously (e.g., if two drug classes are available for empiric therapy of certain infections, treat half of the patients with one drug class and half with the other) will reduce resistance more effectively than cycling under a broad range of conditions (*19*). This suggests that such mixed regimens would be good candidates for comparison with cycling in controlled trials.

As a second example, levels of resistance in hospital-acquired pathogens may change rapidly within a matter of weeks or months after changes in antimicrobial use. By contrast, studies of reductions in antimicrobial use in communities have shown slow and equivocal effects on resistance in community-acquired pathogens (*55*). Mathematical models suggest that, in communities, the key factor driving the change in resistance levels may be the "fitness cost" of resistance, i.e., resistance will decline after a reduction in antimicrobial use if resistant organisms in untreated patients are at a disadvantage for transmission or persistence (*19*,*50*,*56*,*57*). This cost may be small in many bacteria, accounting for the slow response (*55*,*58*). In contrast, a model indicates that, in hospitals, changes in resistance may be driven primarily by the admission of new patients who often bring with them drug-susceptible flora, and this may rapidly "dilute" levels of resistance in the absence of continuing selection by antibiotics (*59*). If correct, this explanation suggests that the success of antimicrobial control measures should be evaluated differently for hospitals and for communities.

The use of mathematical models, and more generally the attempt to predict the relative merits of different interventions, will depend on an improved understanding of the mechanisms of antibiotic selection in particular organisms. For example, two recently published models for the nosocomial spread of resistant pathogens made contrasting assumptions about whether antimicrobial treatment increased an patient’s susceptibility to colonization only during treatment (*60*) or for a period following treatment (*59*), and about the importance of colonization with drug-susceptible strains in protecting against acquisition of resistant ones. As a result of these differences in assumptions, predictions differed in important ways: one model suggested that reduction of antibiotic use would be a comparatively poor intervention when endemic transmission is high and that resistant organisms could persist endemically even in the absence of input from admitted patients or antibiotic selection (*60*). The other model predicted rapid declines in the level of resistance when use is reduced, and a more complicated relationship between the effectiveness of interventions and the level of transmission within the hospital (*59*). Testable predictions will permit the evaluation of different models for particular settings and provide a basis for refining the assumptions of these models.

## Conclusion

The relationship between antibiotic usage and antibiotic resistance for many types of pathogens is largely mediated by indirect effects or population-level selection. When resistant and susceptible organisms compete to colonize or infect hosts, and use of an antibiotic has a greater impact on the transmission of susceptible bacteria than resistant ones, then increasing use of the antibiotic will result in an increase in frequency of organisms resistant to that drug in the population, even if the risk for treated patients is modest. Antimicrobial use and patient-to-patient transmission are not independent pathways for promoting of antimicrobial resistance, rather they are inextricably linked.

Study designs to assess the effect of antimicrobial use on resistance should reflect these diverse pathways of direct and indirect effects. Estimates of direct effects of antimicrobial use on treated patients will be most informative if clinical cultures are combined with measurements of colonization. Use of time-to-event (e.g., Cox proportional hazards) models provides a natural way of controlling for the patient’s length of stay when assessing the effect of treatment on acquisition of resistant organisms. Analyses that control for a person's exposure to other patients carrying resistant organisms will help to capture the effect modification because of varying transmission pressures during a study. Inclusion of data on antimicrobial use by the group to which others are exposed (siblings, fellow patients on a hospital unit, total use in a community) and to individual-level data will provide one method of estimating both direct and indirect effects of antibiotic use. Nonindependence of individual outcomes makes the interpretation of intervention studies problematic unless measures are taken to account for this nonindependence; cluster-randomized studies, used in other areas of infectious disease epidemiology, are an excellent solution to this problem. We have commented elsewhere on other aspects of study design for antimicrobial resistance, notably the importance of control group selection (*7*,*61*,*62*).

Understanding in detail, for each pathogen, the mechanisms by which antimicrobial use selects for antimicrobial resistance in treated patients and in the population is of more than academic importance. For practitioners, these mechanisms matter for making well-informed decisions about the design of treatment protocols, the choice of antibiotics and doses for particular indications. For policymakers, these issues have direct bearing on the design of campaigns to encourage more rational antibiotic use and on the priorities in regulating the use of antimicrobial agents for human and animal use (*63*,*64*).
